# Quality of life and its social determinants for patients with schizophrenia and family caregivers in Cambodia

**DOI:** 10.1371/journal.pone.0229643

**Published:** 2020-03-04

**Authors:** Toshiyuki Marutani, Sotheara Chhim, Akihiro Nishio, Akiko Nosaki, Yasuko Fuse-Nagase

**Affiliations:** 1 Health Support Center, Tokyo Institute of Technology, Yokohama, Japan; 2 Supporters for Mental Health, Tokyo, Japan; 3 Transcultural Psychosocial organization Cambodia, Phnom Penh, Cambodia; 4 Health Administration Center, Gifu University, Gifu, Japan; 5 Psychiatric and Mental Health Nursing, Graduate School of Nursing, Chiba University, Chiba, Japan; 6 University Health Center, Ibaraki University, Mito, Japan; KHANA, CAMBODIA

## Abstract

Due to inadequate human and financial resource support, the development of mental health services in Cambodia has been undertaken by various non-governmental organizations (NGOs). Schizophrenia is the most common functional psychotic disorder, causing severe and chronic symptoms, and the programs provided by the NGOs should have enhanced the quality of life (QoL) of patients and their caregivers; however, epidemiological research, which is a driving force behind the recognition of mental health as a global public health concern, is lacking for schizophrenia in Cambodia. This study therefore aimed to create QoL evaluation questionnaires available in Khmer (the Cambodian language) for patients with schizophrenia and family caregivers, and to identify the social determinants and predictors of their QoL. This cross-sectional study recruited 59 patients and 59 caregivers attending three clinics operated by two NGOs: the Transcultural Psychosocial Organization (TPO) Cambodia and the Supporters for Mental Health (SUMH) Cambodia. We conducted linguistic validation of the Schizophrenia Quality of Life Questionnaire 18-item version (S-QoL 18) and the Schizophrenia Caregiver Questionnaire (SCQ), then analyzed correlations between the QoL dimensions and socio-demographic factors. The main findings of this study were as follows: 1) the newly created Khmer versions of S-QoL 18 and SCQ are relatively good psychometric tools that are suitable for research to identify patients’ and caregivers’ needs to improve their QoL; and 2) engaging in paid work or being of the post-Khmer Rouge generation results in higher QoL for patients, but having low household economic status or being affected by chronic disease leads to lower QoL for family caregivers. These findings are useful for enabling community mental health professionals and aid organizations to create programs to lessen the patient and caregiver burden in Cambodia. Further research is necessary to develop practical projects that will improve patients’ and caregivers’ QoL in various clinical settings in Cambodia.

## Introduction

In Cambodia, Pol Pot’s Khmer Rouge (KR) regime (Democratic Kampuchea, 1975–1979) devastated the overall health care system, and it has been reported that none of the psychiatrists, or patients with psychiatric disorders, and survived that era [1]. Since the resumption of psychiatric services in Cambodia in 1994, significant advances have been made in the field, but psychiatric services are still extremely inconsistent [1, 2]. While the average economic growth rate was 7.7% between 1995 and 2017 [3], the annual mental health budget is estimated to be only approximately 0.02% of the national health budget [4]. Cambodia’s current strategic health plan includes increased coverage and access to primary and complementary mental health services as one of its objectives; however, it is only partially and inadequately implemented in practice [4, 5]. Currently, there are 56 psychiatrists in the country, with a ratio of 0.33 psychiatrists per 100,000 people [1]. There are no mental health supported accommodation services [6] or acute psychiatric units for short-term hospitalization, such as those available in developed countries [7]. The total number of inpatient beds was 15 for the whole country in 2011, and the current situation is unclear [1, 8]. Clearly, community and outpatient care for patients with psychiatric disorders is essential.

Due to inadequate human and financial resources, the development of community mental health services in Cambodia has been undertaken by diverse non-governmental organizations (NGOs), which provide different community mental health services in various Cambodian provinces [1]. Transcultural Psychosocial Organization (TPO) Cambodia is the leading and most consistent NGO in this field. It runs numerous, and multifaceted community mental health programs and provides regular outpatient consultation services in its treatment center in Phnom Penh and, less frequently, in clinics in its five branch offices [1]. Supporters for Mental Health (SUMH) Cambodia is an organization that in another one which operates in Siem Reap Province. Its principal aim was to build a mental health service system in the Siem Reap Province following a training program and a mental health clinic run by Harvard School of Public Health which terminated at the end of 1999 [1, 9, 10]. SUMH Cambodia based their projects on a 2001–2003 population survey of mental health-related situations in two communities in Siem Reap Province [11]. Juntapim and Nuntaboot (2018) [12] reported that the community care process should include at least the three following steps: 1) understanding the problems and needs relating to care, risk factors, and solutions; 2) searching for the social capital of community and collaboration to promote the care and quality of life (QoL) of patients with mental health problems; and 3) determining suitable approaches for the solution of health problems and other issues through network development. The development of SUMH Cambodia’s mental health care system was, by and large, aligned with these aspects. SUMH Cambodia staff understood the problems and needs for care; discussed solutions; collaborated to encourage people living around the patients, and those engaged in traditional and regional medicine, to work together with the psychiatric outpatient clinics; and promoted cooperation with other NGOs and public administration services [13, 14].

These programs should have enhanced the QoL of patients and their caregivers; however, epidemiological research documenting the considerable health burden caused by mental disorders, which is a driving force behind the recognition of mental health as a global public health concern, is lacking in Cambodia [4]. Notable exceptions concern post-traumatic stress disorder and other trauma-related disorders induced by Pol Pot’s KR regime [15–17]. This critical era psychologically affected Cambodian individuals over the long term, and it is one of the primary reasons for the generation gap between those who lived through the KR regime and those who learned of it as a historical horror story in school [18, 19]. We assumed that this past trauma could affect the QoL of those who survived the KR era. Other than trauma-related disorders, more general psychiatric and psychological problems, including schizophrenia, are largely undocumented [4]. To the best of our knowledge, the QoL of patients with schizophrenia and their family caregivers in Cambodia has not been investigated.

Schizophrenia is the most stigmatizing and most common functional psychotic disorder, causing an array of severe and chronic symptoms, such as delusions, hallucinations, disorganized speech or behavior, and impaired cognitive ability [20–22]. Of 683 patients admitted to the TPO Cambodia clinic in 2017, 16% were patients with schizophrenia [23]. Of 973 patients admitted to the SUMH Cambodia rehabilitation and consultation service centers in 2016, 32.1% were patients with schizophrenia [24]. The early onset of the disease, along with its chronic nature, makes it a disabling disorder that significantly affects the QoL of patients and their family caregivers [20, 25]. Strong family involvement in mental health care is widespread in Asia; more than 70% of patients with schizophrenia depend on their families, in contrast to about 25–50% in Western countries [26, 27]. Moreover, the average duration of untreated mental illness (DUP) in patients with schizophrenia in Cambodia is prolonged, such as 47 months in a previous report [28]. Taken together, patients and their family caregivers have already suffered a long-term disease burden and stigmatization by their first visit to psychiatric medical services [4]. After the treatment starts, focusing on the QoL of patients with schizophrenia beyond the clinical improvement of symptoms is essential [29, 30]. Health-related QoL is defined as an individual’s perception of their position in life, in the context of their culture and value systems, regarding their goals, expectations, standards, and concerns [31]. Caregivers face considerable burdens and challenges relating to their QoL when they support individuals with schizophrenia [32, 33], so improvement in the caregiver’s QoL has an impact on patient’s psychotic symptoms and QoL [34]; thus, focusing on the QoL of family caregivers is crucial for evaluating the critical outcomes of community care.

In order to evaluate the QoL of patients with schizophrenia, a disease-specific questionnaire is desirable, and the Schizophrenia Quality of Life Questionnaire (S-QoL) was thus developed [35]. For the family caregivers of patients with schizophrenia, the Schizophrenia Caregiver Questionnaire (SCQ) was developed from the Zarit Burden Interview, which was widely used, but intended to assess the burden of caregivers for patients with senile dementia [36–38]. Both were unavailable in Khmer (the Cambodian language). The QoL-related questionnaires already available in Khmer were the Short Form-12 Health Survey version 1 (SF-12 v1) and the 10-item Connor–Davidson Resilience Scale (CD-RISC-10). The former is used for physical and mental health status self-evaluation, and the latter is used for evaluation of resilience (the personal qualities that enable a person to thrive in the face of adversity) [39]. The physical and psychological health status contributes to the QoL of patients with schizophrenia [40], and QoL and resilience are moderately correlated in both patients and caregivers [41, 42]; therefore, these scales were used to validate the newly-developed Khmer versions of the QoL questionnaires.

The current study aimed to 1) create and validate QoL evaluation questionnaires in Khmer for patients with schizophrenia and family caregivers in Cambodia and 2) identify correlations between socio-demographic factors and the dimensions of QoL questionnaires to find the social determinants of improving or worsening QoL in Cambodian clinical settings.

## Materials and methods

### Participants

We purposively recruited patients and family caregivers when they visited the SUMH Cambodia psychiatric rehabilitation center in Siem Reap and the TPO Cambodia clinics in Phnom Penh and Kampong Cham for their regular follow-up rehabilitation or consultation appointments. The patients were diagnosed with schizophrenia by psychiatrists in the Psychiatry Department of Siem Reap Provincial Hospital or TPO Cambodia clinics, based on the International Classification of Diagnosis, 10th edition (ICD–10), or the Diagnostic and Statistical Manual of Mental Disorders, 4th edition (DSM–IV). All of the patients and family caregivers were aged ≥ 18 years. The clinical staff determined the patients’ capacity to give their consent by their communication skills and degree of understanding of the description of the research procedure. The patients, who could respond to the questions with a little help from their caregivers, were also invited to participate in the study. The caregivers, a fair number of whom attended without the patients present, were also invited. Since there were only 24 patient–caregiver pairs, comparison between pairs was excluded due to the small sample size. This survey was combined with qualitative analysis of a semi-structured interview study, which lasted 1 to 1.5 h each, but at that point, some of those who traveled to the clinic over long distances or were worried about their concentration declined to participate in the study.

This cross-sectional study was conducted from September 2017 to March 2018 and included 118 Cambodian participants, of whom 59 were patients and 59 were family caregivers. The numbers of patients and family caregivers in Phnom Penh, Kampong Cham, and Siem Reap were 36, 18, and 5, and 25, 16, and 8, respectively. The participants in each clinic came from both urban and rural areas.

### Materials and procedures

We collected socio-demographic data and conducted a linguistic validation process on the S-QoL 18-item version (S-QoL 18) and SCQ to produce the first Khmer versions, and used the Khmer versions of the SF-12 v1 and CD-RISC-10 to validate them. The generation gap between the pre- and post-KR era people was evaluated by dividing the subjects into those who were born before or during the KR regime (aged ≥ 38 years) and those born after. For linguistic validation, two bilingual Cambodian translators performed the forward translation into Khmer, and these translations were reconciled by co-author Chhim to create the first Khmer version. In the next step, the Khmer version was back-translated by a third bilingual Cambodian translator into English, and the two English versions were compared by Chhim to finalize the second Khmer version for the present study. We then assessed internal consistency reliability by calculating the Cronbach’s alpha coefficient [43]. Concurrent validity was assessed using Pearson’s correlation coefficients between each domain of S-QoL18 or SCQ and the validation questionnaires (SF-12 v1 and CD-RISC-10). We also translated the 6-item version of the Positive and Negative Syndrome Scale for Schizophrenia (PANSS-6) [44]. Co-author Chhim conducted forward translation to develop the Khmer version of PANSS-6 to evaluate the severity of the patients’ symptoms. The linguistic validation was not conducted for PANSS-6, because the translated sentences were used by the interviewers as a tool to evaluate the severity of patients’ symptoms, and not used as questionnaires to the patients. The first author trained the staff of SUMH Cambodia to rate PANSS-6, while psychiatrists rated PANSS-6 in the TPO Cambodia clinics [45]. Given the literacy rate of Cambodia (74%, 2009–2014) [46], we conducted interviews using the questionnaires, training and supervising the staff of SUMH Cambodia and TPO Cambodia as interviewers.

### Short Form-12 Health Survey version 1 (SF-12 v1)

SF-12 v1 is the first version of SF-12: a 12-item version of the 36-item Short Form Health Survey (SF-36), which is a widely-used self-perceived health status questionnaire designed for both general and specific populations [47]. Each item is rated on a Likert-type scale. We used the weighted and normed scoring rules recommended by the SF-12 developers (Ware JE Jr et al.) for the physical and mental health component summary scores (PCS and MCS, respectively), with lower scores indicating higher disability [48]. Since PCS and MCS normative data for the Cambodian population was unavailable, we used normative data based on the US population in 1990, which Sonis et al. [15] had applied to the Cambodian population and which showed excellent reliability.

### The 10-item Connor-Davidson Resilience Scale (CD-RISC-10)

CD-RISC-10 is a 10-item short version of the 25-item CD-RISC: an excellent, widely-used psychometric scale for measuring resilience in the adults [49]. Items were rated on a 5-point Likert scale relating the past month’s condition and combined into a single scale, with a higher score indicating higher resilience [50]. The CD-RISC-10 Khmer version showed excellent reliability and validity when used for Cambodian adolescents [51], and permission for its use was obtained from the developers (Davidson JRT and Connor KM) in May 2017.

### Schizophrenia Quality of Life Questionnaire 18-item Version (S-QoL 18)

The S-QoL 18 is the short version of a 41-item French self-administered multidimensional QoL questionnaire concerning the present circumstances and designed for patients with schizophrenia [35, 52]. It comprises 18 items describing eight dimensions: psychological well-being (PsW), self-esteem (SE), family relationships (RFa), relationships with friends (RFr), resilience (RE), physical well-being (PhW), autonomy (AU), and sentimental life (SL), together with the index (total score). The dimension scores were calculated by summing the self-rated 5-point Likert scale scores and transferring them linearly onto a scale of 0–100, with higher scores indicating higher QoL. We obtained permission to use and translate the English version of SCQ from the Mapi Research Institute in July 2017.

### Positive and Negative Syndrome Scale for Schizophrenia 6-item version (PANSS-6)

The original version of PANSS is a 30-item, 7-point (1–7) clinician-rated scale that was specifically developed to assess psychotic symptoms in individuals with schizophrenia [53]. Although it is the most widely-used measure of schizophrenia severity, it is too lengthy for research purposes, especially for research using other measures and interviews, such as the present study. Of the shorter versions of PANSS, developed for use in both research and clinical settings, we selected the shortest 6-item version proposed by Østergaard et al. [44]. Although it was applied only to hospitalized acute-phase patients and could capture higher symptom reduction and remission rates compared with controls, we used it for the present study, together with a semi-structured interview study for qualitative analysis (unpublished data at present), which required a rapid evaluation of patients’ symptom severity. 3-item positive symptom scores, 3-item negative symptom scores, and the total score were calculated.

### Schizophrenia Caregiver Questionnaire (SCQ)

The 32-item SCQ was developed from the ZBI to assess the burden on caregivers of patients with schizophrenia [37], which is rated on an 11-point Likert scale for the past four weeks, and each score is converted to 5-point item scores. The SCQ consists of two parts; a “humanistic impact—total score (HI-TS)” supra-domain to assess the direct impact on the individual caregiver, composed of four subdomains for physical, emotional, social, and daily life aspects (HI-P, HI-E, HI-S, HI-DL, respectively), and another part to assess all other aspects of the caregiver experience and indirectly reflect their personal impact, composed of eight domains such as “exhaustion with caregiving (EC)”, “patient dependence (PD)”, “worries for the patient (WP)”, “perception of caregiving (PC)”, “feeling alone (FA)”, “financial dependence of the patient (FDP)”, “financial impact of caregiving (FIC)”, and “overall difficulty of caregiving (ODC)”. Each domain score is calculated as a simple sum of the item scores, transferred linearly onto a scale of 0–100, with higher scores indicating lower QoL [37, 38].

### Data analysis

We calculated the questionnaire scores according to the structure of the original version. We assessed the internal consistency reliability by calculating the Cronbach’s alpha coefficient, with a coefficient of at least 0.7 expected for each dimension [43]. When we assessed the concurrent validity using Pearson’s correlation coefficients between each domain of S-QoL18 or SCQ and the validation questionnaires, an absolute coefficient value between 0.4 and 0.7 was expected for a moderate correlation [54]. Parallel analysis based on principal component analysis was conducted to assess the structure of the Khmer versions of S-QOL 18 and SCQ. To evaluate the relationships between the socio-demographic factors and the questionnaire scores, correlation analysis, unpaired *t*-tests, or Mann-Whitney’s *U* test were used. We then further analyzed all the significant and nearly-significant factors associated with the overall QoL scores, using multiple linear regression analysis with dummy variables for nominal variables. All the tests were two-tailed and statistical significance was defined as *p* < 0.05. The statistical analyses were performed using IBM SPSS Statistics for Macintosh, Version 25.0, except for the parallel analysis, which was performed using R version 3.5.2 with “psych package” version 1.8.12 [55, 56].

### Ethical issue

This study protocol was approved by the Tokyo Institute of Technology Ethics Committee for Human Research (2016078) and the National Ethics Committee for Health Research in Cambodia (031NECHR). Before commencing the survey, written informed consent was obtained from the participants and the study objectives and its voluntary participation were explained.

## Results

### Socio-demographic data

The socio-demographic data is presented in Table 1. The mean patients’ age was significantly lower than that of the family caregivers (p = 0.001). Educational level, indicated by years of schooling and literacy, were significantly higher in the caregivers than in the patients (p = 0.026). 93.2% of the patients used the typical antipsychotics haloperidol and/or chlorpromazine; the only atypical one was risperidone, and the chlorpromazine-equivalent dose of the medication for the patients is also shown in Table 1. Among caregiver participants, 63.3% recognized themselves as the principal caregivers, and 71.1% of the principal caregivers were parents of the patients.

**Table 1:** Description of baseline characteristics of participants (Patients n = 59, Caregivers n = 59).

		Patients	Caregivers
Age (years), mean (SD)		39.4 (11.7)	47.9 (14.7)
Generation, *n*(%)	Pre-KR generation	31 (52.5)	41 (69.5)
	Post-KR generation	28 (47.5)	18 (30.5)
Gender, *n*(%)	Male	24 (40.7)	22 (37.3)
	Female	35 (59.3)	37 (62.7)
Marital status, *n*(%)	Married	23 (39.0)	42 (71.2)
	Separated	2 (3.4)	4 (6.7)
	Divorced	6 (10.2)	4 (6.8)
	Widowed	4 (6.8)	9 (15.3)
	Never married	24 (40.7)	4 (6.8)
Education (years of schooling), mean(SD)		5.8 (4.7)	9.5 (12.9)
Literacy, *n*(%)	Literate	40 (67.8)	50 (84.7)
	Illiterate	19 (32.2)	9 (15.3)
Occupation, *n*(%)	Agricultural	14 (23.7)	17 (28.8)
	Employee	9 (15.3)	10 (16.9)
	Family operated or independent	10 (16.9)	18 (30.5)
	Housewife	4 (6.8)	5 (8.5)
	Student	0 (0.0)	0 (0.0)
	Unemployed	20 (33.9)	9 (15.3)
Economic status, *n*(%)	Sufficient	19 (32.2)	18 (30.5)
	Insufficient	37 (62.7)	33 (55.9)
Residence, *n*(%)	Urban	28 (47.5)	28 (47.5)
	Rural	30 (50.8)	31 (52.5)
Religion, *n*(%)	Buddism	55 (93.2)	59 (100.0)
	Christianity	4 (6.8)	0 (0.0)
No of person household, mean (SD)		5.4 (2.5)	5.4 (2.4)
Chlorpromazine equivalent dose (mg), mean (SD)		397.0 (360.7)	435.4 (395.2)^a^
Duration of illness (years), mean (SD)		9.6 (8.4)	8.0 (6.4)^a^
Duration of untreated psychosis (DUP) (years), mean (SD)		3.8 (5.7)	2.0(3.0)^a^
Chronic physical complications, Chronic disease, *n*(%)		15 (25.4)	30 (50.8)
Duration of chronic complications (years), mean (SD)		6.9 (6.5)	8.0 (8.8)
Time of caregiving (hours/day), mean (SD)			5.2 (5.9)
Kinship, n(%)	Parent		32 (54.2)
	Spouse		6 (10.2)
	Sibling		9 (15.3)
	Offspring		4 (6.8)
	Other relatives		6 (10.2)
Principal Caregiver, *n*(%)			38 (63.3)

*Note*: KR = Khmer Rouge

^a^Data of the patient of the family participant

### Scale scores and internal consistency reliability

The Cronbach’s alpha coefficients for SE, RFa, RE, PhW, AU, index of S-QoL 18, HI-TS and its subdomains, and the total score and WP of SCQ, SF-12 v1, CD-RISC-10, and PANSS-6 scores, were over the recommended threshold of 0.70 (0.731–0.962), indicating good internal consistency reliability [25] (Table 2). The Cronbach’s alpha coefficients for PsW, RFr, SL on the S-QoL 18 and EC, PD, and PC on the SCQ, did not reach the acceptable range for reliability. The SF-12 PCS and MCS, and CD-RISC-10 scores did not differ significantly between patients and caregivers (Table 2).

**Table 2:** Description of S-QoL18, SF-12, CD-RISC 10, PANSS-6 scores and internal consistency reliability.

	Dimensions	(Item No)	# of item	Subject	Mean Score (SD)	*p*	Cronbach's alfa
S-QoL18	PsW	16,17,18	3	Patient	60.6(25.4)		0.527
	SE	1,4	2	Patient	65.9(25.5)		**0.731**
	RFa	10,11	2	Patient	76.7(25.6)		**0.869**
	RFr	12,13	2	Patient	55.9(25.3)		0.548
	RE	2,3,7	3	Patient	67.8(25.2)		**0.775**
	PhW	8,9	2	Patient	55.7(31.1)		**0.882**
	AU	5,6	2	Patient	67.8(28.0)		**0.861**
	SL	14,15	2	Patient	52.8(30.4)		0.621
	Index (total)	1_18	18	Patient	62.9(18.5)		**0.878**
SCQ	HI-TS		17	Caregiver	39.9(25.8)		**0.952**
	HI-P	10,24,29	3	Caregiver	38.3(31.3)		**0.833**
	HI-E	4–6,25,30,31	6	Caregiver	41.5(28.0)		**0.903**
	HI-S	7,12,13	3	Caregiver	36.3(28.1)		**0.759**
	HI-DL	2,3,11,17,32	5	Caregiver	41.0(25.7)		**0.823**
	EC	16,18	2	Caregiver	34.3(28.9)		0.637
	PD	1,14	2	Caregiver	42.6(30.2)		0.544
	WP	8,27,28	3	Caregiver	53.5(26.3)		**0.763**
	PC	20,21	2	Caregiver	58.9(25.3)		0.427
	FA	23	1	Caregiver	38.6(38.1)		NA
	FDP	9	1	Caregiver	60.6(39.7)		NA
	FIC	15	1	Caregiver	54.2(36.6)		NA
	ODC	22	1	Caregiver	44.1(35.8)		NA
	SCQ total		32	Caregiver	42.8(23.4)		**0.962**
SF-12v1	PCS		12	Patient	45.0(10.4)	0.09	**0.785**
			12	Caregiver	42.1(8.2)		**0.781**
	MCS		12	Patient	42.5(9.5)	0.88	**0.785**
			12	Caregiver	38.5(8.5)		**0.781**
CD-RISC-10			10	Patient	22.0(6.9)	0.07	**0.839**
			10	Caregiver	24.4(7.0)		**0.862**
PANSS-6	Positive		3	Patient	6.4(3.5)		**0.798**
	Negative		3	Patient	6.6(4.2)		**0.882**
	Total		6	Patient	13.0(6.7)		**0.849**

*Note*: Cronbach's alfa; in bold, above the recommended threshold of 0.70; PsW = Psychological well-being

SE = Self-esteem; RFa = Family relationships; RFr = Relationshios with friends; RE = Resilience

PhW = Physical well-being; AU = Autonomy; SL = Sentimental life; HI-TS = Humanistic impact—Total Score

HI-P = Humanistic impact—Physical; HI-E = Humanistic impact—Emotional; HI-S = Humanistic impact—Social

HI-DL = Humanistic impact—Daily life; EC = Exhaustion with caregiving; PD = Patient dependence

WP = Worries for the patient; PC = Perception of caregiving; FA = Feeling alone

FDP = Financial dependence of the patient; FIC = Financial impact of caregiving

ODC = Overall difficulty of caregiving; PCS = Physical component summary; MCS = Mental component summary

The parallel analysis, based on principal component analysis, showed that there were two, one, and one adequate number of factors for the S-QoL 18, SCQ total, and SCQ HI-TS Khmer versions, respectively, which did not have the same structure as the original versions.

### Concurrent validity of S-QoL and SCQ Khmer version

Pearson’s correlation coefficients between the S-QoL 18 and SCQ domains and the validation questionnaires or scales are shown in Table 3. The S-QoL 18 index and domains, except for PsW and RFa, were moderately correlated with SF-12 MCS and CD-RISC-10 (|*r*| = 0.417–0.637). These domains and SL were moderately correlated with the PANSS scores (|*r*| = 0.415–0.572). SF-12 PCS was moderately correlated only with PhW. The PsW and RFa dimensions were uncorrelated or weakly correlated with all the validation scale scores. For family caregiver measurements, HI-TS, HI-P, HI-E, HI-DL, PD, WP, FIC, ODC, and the total score were moderately correlated with SF-12 PCS (|*r*| = 0.416–0.560). Only HI-S was moderately correlated with the SF-12 MCS (*r* = -0.426). All the domains and the total SCQ score were uncorrelated or weakly correlated with CD-RISC-10.

**Table 3:** Correlations between S-QoL 18 or SCQ scores and SF-12, CD-RISC 10, and between S-QoL 18 and PANSS-6 scores.

		S-QoL18 domains													
		PsW	SE	RFa	RFr	RE	PhW	AU	SL	Index					
SF-12	PCS	0.125	0.301*	0.062	0.269*	0.174	0.513**	0.213	0.208	0.350**					
	MCS	0.081	0.610**	0.375**	0.430**	0.493**	0.547**	0.431**	0.348**	0.609**					
CD-RISC-10		0.274*	0.637**	#####	0.492**	0.545**	0.421**	0.417**	0.354**	0.571**					
PANSS-6	Positive	0.017	-0.346**	#####	-0.372**	-0.327*	-0.278*	-0.505**	-0.377**	-0.439**					
	Negative	0.127	-0.409**	#####	-0.359**	-0.544**	-0.24	-0.486**	-0.343**	-0.438**					
	Total	0.089	-0.440**	#####	-0.422**	-0.513**	-0.298*	-0.572**	-0.415**	-0.507**					
		SCQ domains													
		HI-TS	HI-P	HI-E	HI-S	HI-DL	EC	PD	WP	PC	FA	FDP	FIC	ODC	SCQ total
SF-12	PCS	-0.497**	-0.543**	-0.416**	-0.300*	-0.560**	-0.25	-0.443**	-0.412**	-0.21	-0.294*	-0.284*	-0.442**	-0.470**	-0.501**
	MCS	-0.394**	-0.339**	-0.328*	-0.426**	-0.391**	-0.321*	-0.284*	-0.285*	-0.11	-0.295*	-0.16	-0.377**	-0.352**	-0.398**
CD-RISC-10		-0.318*	-0.333*	-0.282*	-0.267*	-0.301*	-0.25	-0.23	-0.13	-0.12	-0.21	0.174	-0.14	-0.2	-0.297*

*Note*: Pearson's correlation coefficients; In bold, 0.40 < absolute value of correlations

* p < 0.05

** p < 0.01; Abbreviations: see the note on Table 2.

### Socio-demographic factors associated with scale scores

The univariate analysis of the associations between the socio-demographic data, medication use, and measures of patients is shown in Table 4. In patients, the scale scores for gender, age, education, and literacy were not significantly different, except for literacy and the SL domain. The post-KR generation showed higher scores in the S-QoL 18 for RFa and the index, and more severe negative symptoms. If the patients had a paid occupation, the S-QoL 18 RE, AU, SL, and SF-12 PCS scores were higher, and all the PANSS-6 scores were lower, than for those who performed unpaid work or did not work. Economic status was associated with PhW and the SF-12 MCS, but chronic physical complications were associated only with the MCS. A higher chlorpromazine-equivalent dose was mildly correlated to PhW and all the PANSS-6 scores. Marital status (married or others), residence (urban or rural), number of people per household, duration of patient’s illness, and DUP did not affect any QoL or validation questionnaires (Table in S1 Table).

**Table 4:** Socio-demographic factors of patients associated with S-QoL 18, SF-12, CD-RISC 10, and PANSS-6.

			Gender		Age	Generation		Education	Literacy		Occupation		Economic status		Physical complications		CP
			Male	Female	(years)	Pre-KR	Post-KR	(years of schooling)	Litterate	Illitterate	Paid	Unpaid	Sufficient	Insufficient	Yes	No	equivalent dose (mg)
S-QoL 18	PsW	M (SD) or *r*	63.5 (26.6)	58.6 (24.8)	-0.211	54.8 (23.3)	67.0 (26.6)	0.274	64.2 (23.7)	53.1 (27.8)	59.8 (26.0)	61.8 (26.1)	68.4 (25.2)	56.1 (25.3)	66.7 (15.3)	59.1 (27.9)	0.154
		*p*	0.465		0.108	0.067		**0.037**	0.118		0.780		0.089		0.204		0.244
	SE	M (SD) or *r*	65.1(25.3)	66.4(26.0)	0.028	64.1(25.4)	67.9(26.0)	-0.056	65.3(28.5)	67.1(18.3)	70.5(23.8)	60.9(26.9)	72.4(26.2)	64.9(24.6)	59.8(26.0)	68.2(25.5)	-0.228
		*p*	0.847		0.833	0.578		0.674	0.803		0.164		0.295		0.292		0.082
	RFa	M (SD) or *r*	78.1 (27.6)	75.7 (24.4)	-0.106	**69.4 (30.6)**	**84.8 (15.3)**	-0.057	75.9 (26.6)	78.3 (23.9)	78.8 (26.6)	76.0 (24.4)	80.3 (16.8)	76.4 (29.6)	81.3 (20.1)	75.9 (27.1)	-0.116
		*p*	0.725		0.424	**0.019**		0.670	0.745		0.691		0.597		0.496		0.381
	RFr	M (SD) or *r*	58.3 (27.0)	54.3 (24.2)	-0.023	52.8 (24.5)	59.4 (26.0)	-0.011	53.1 (25.9)	61.8 (23.4)	59.8 (27.2)	52.1 (21.4)	58.6 (25.7)	56.1 (24.6)	55.4 (21.8)	57.1 (25.9)	-0.256
		*p*	0.550		0.864	0.324		0.937	0.218		0.251		0.727		0.821		0.050
	RE	M (SD) or *r*	66.7 (27.8)	68.6 (23.6)	-0.066	65.9 (26.1)	69.9 (24.4)	-0.105	67.3 (24.4)	68.9 (27.3)	**75.5 (25.2)**	**59.0 (20.8)**	73.7 (22.4)	66.9 (25.6)	66.1 (23.9)	68.9 (25.8)	-0.282
		*p*	0.778		0.620	0.539		0.432	0.825		**0.011**		0.333		0.714		**0.030**
	PhW	M (SD) or *r*	60.4 (33.7)	52.5 (29.2)	-0.258	48.8 (28.8)	63.4 (32.3)	-0.060	52.8 (32.5)	61.8 (27.8)	57.2 (33.8)	54.2 (28.0)	**67.8 (28.4)**	**50.3 (31.7)**	50.9 (32.3)	58.0 (30.9)	**-0.430**
		*p*	0.341		0.049	0.071		0.655	0.301		0.721		**0.049**		0.464		**0.001**
	AU	M (SD) or *r*	67.2 (31.7)	68.2 (25.6)	-0.081	63.3 (29.6)	72.8 (25.7)	-0.285	64.1 (29.3)	75.7 (23.7)	**75.0 (30.1)**	**59.4 (23.1)**	71.7 (27.9)	66.6 (29.0)	66.1 (27.9)	68.8 (28.5)	-0.366
		*p*	0.891		0.543	0.197		**0.030**	0.112		**0.038**		0.526		0.759		**0.004**
	SL	M (SD) or *r*	59.4 (28.1)	48.2 (31.5)	-0.068	47.2 (32.2)	58.9 (27.6)	-0.235	**47.2 (30.0)**	**64.5 (28.6)**	**63.6 (28.9)**	**41.1 (26.7)**	53.9 (32.6)	52.4 (30.5)	46.4 (33.4)	55.4 (29.6)	-0.216
		*p*	0.169		0.609	0.140		0.076	**0.040**		**0.004**		0.858		0.342		0.101
	Index (total)	M (SD) or *r*	64.8 (20.8)	61.6 (16.9)	-0.148	**58.3 (19.0)**	**68.0 (16.8)**	-0.108	61.2 (18.6)	66.4 (18.2)	67.5 (19.9)	58.1 (14.6)	68.3 (19.7)	61.2 (17.8)	61.6 (18.1)	63.9 (18.6)	-0.329
		*p*	0.508		0.262	**0.043**		0.420	0.321		0.054		0.175		0.681		**0.011**
SF12	PCS	M (SD) or *r*	47.6 (9.7)	43.3 (10.7)	-0.108	45.1 (11.7)	44.9 (8.9)	-0.038	44.4 (11.2)	46.4 (8.5)	**47.3 (9.1)**	**41.6 (11.7)**	45.6 (11.0)	44.5 (10.6)	39.0 (13.5)	46.9 (8.7)	-0.036
		*p*	0.125		0.414	0.936		0.778	0.481		**0.045**		0.729		0.056		0.786
	MCS	M (SD) or *r*	43.7 (9.3)	41.6 (9.6)	-0.175	40.5 (10.1)	44.6 (8.3)	0.125	43.4 (9.6)	40.6 (9.1)	42.1 (9.3)	42.8 (9.8)	**47.2 (10.0)**	**40.3 (8.7)**	**38.0 (10.9)**	**43.9 (8.7)**	-0.210
		*p*	0.435		0.186	0.090		0.348	0.291		0.781		**0.010**		**0.040**		0.111
CD-RISC-10		M (SD) or *r*	22.3 (6.8)	21.9 (7.1)	-0.127	23.2 (6.9)	20.8 (6.8)	0.008	21.7 (7.1)	22.7 (6.7)	22.9 (6.8)	21.2 (6.9)	23.4 (7.0)	21.7 (7.0)	19.7 (5.7)	22.8 (7.2)	-0.249
		*p*	0.815		0.339	0.191		0.955	0.623		0.347		0.383		0.152		0.057
PANSS-6	Positive	M (SD) or *r*	6.3 (2.6)	6.5 (4.1)	-0.104	6.1 (3.4)	6.8 (3.8)	0.208	6.9 (4.0)	5.5 (2.2)	**5.5 (3.3)**	**7.7 (3.7)**	6.8 (4.2)	6.1 (3.2)	7.0 (4.1)	6.2 (3.4)	**0.426**
		*p*	0.849		0.433	0.437		0.117	0.174		**0.024**		0.498		0.471		**0.001**
	Negative	M (SD) or *r*	6.9 (4.1)	6.4 (4.3)	-0.227	**5.4 (3.2)**	**7.9 (4.8)**	0.271	7.3 (4.6)	5.2 (2.7)	**5.2 (3.4)**	**8.6 (4.5)**	6.4 (4.5)	6.3 (3.8)	5.9 (4.1)	6.7 (4.3)	**0.485**
		*p*	0.673		0.084	**0.026**		**0.040**	0.081		**0.002**		0.933		0.482		**<0.001**
	Total	M (SD) or *r*	13.2 (5.1)	12.9 (7.7)	-0.197	11.5 (6.1)	14.7 (7.0)	0.280	14.1 (7.4)	10.7 (4.1)	**10.7 (6.4)**	**16.3 (6.0)**	13.2 (7.9)	12.4 (6.1)	12.9 (7.7)	13.0 (6.5)	**0.412**
		*p*	0.870		0.135	0.067		**0.033**	0.069		**0.001**		0.684		0.954		**0.001**

*Note*: CP = Chlorpromazine; M (SD) = mean (standard deviation); r = Pearson's correlation coefficients; In bold, 0.40 < correlations, p-value; In cold, p < 0.05; Abbreviations: see the note of Table 2

The univariate analysis of the associations between the socio-demographic data, medication use, and measures of family caregivers is shown in Table 5. For family caregivers, female participants showed lower SF-12 MCS and CD-RISC-10 scores, while the SCQ scores were gender-independent. Age and education were not correlated to any scales, and occupation influenced only the SCQ ODC domain and SF-12 MCS. When the economic status of the household was insufficient to meet basic needs, or the family caregivers had chronic disease, were parents of the patient, or were the principal caregivers, the QoL scores were generally lower. The chlorpromazine-equivalent dose for the patients was not correlated to any scores of SCQ, SF-12, or CD-RISC-10. Pre- or post-KR generation, marital status (married or others), residence (urban or rural), duration of caregiving, number of persons per household, duration of patient’s illness, and DUP did not make differences in any QoL or validation questionnaires (Table in S2 Table).

**Table 5:** Socio-demographic factors of family caregivers associated with SCQ, SF-12, and CD-RISC 10.

			Gender		Age	Education	Literacy		Occupation		Economic status		Physical diseases		Kinship		Principal caregiver		CP^b^
			Male	Female	(years)	(years of schooling)	Litterate	Illitterate	Paid	Unpaid	Sufficient	Insufficient	Yes	No	Parent	Other	Yes	No	equivalent dose (mg)
SCQ	HI-TS	M (SD) or *r*	33.7 (19.4)	43.6 (28.6)	0.105	-0.009	**36.4 (23.3)**	**59.0 (31.9)**	37.4 (25.2)	47.9 (27.2)	**26.5 (22.2)**	**47.6 (26.8)**	**47.5 (28.9)**	**29.3 (19.8)**	**47.4(26.1)**	**30.2(23.7)**	44.6 (28.0)	29.0 (21.2)	0.231
		*p*	0.121		0.429	0.948	**0.015**		0.186		**0.006**		**0.008**		**0.013**		0.052		0.086
	HI-P	M (SD) or *r*	31.1 (27.2)	42.6 (33.1)	0.057	-0.203	**34.3 (29.1)**	**60.2 (35.8)**	34.8 (32.2)	49.4 (26.2)	**24.1 (28.7)**	**45.7 (32.9)**	**46.1 (34.9)**	**27.0 (25.5)**	**47.1(31.9)**	**27.0(28.4)**	**45.2 (33.8)**	**21.9 (22.1)**	0.288
		*p*	0.174		0.669	0.123	**0.021**		0.129		**0.023**		**0.023**		**0.016**		**0.005**		**0.031**
	HI-E	M (SD) or *r*	35.6 (21.9)	45.0 (30.1)	0.126	0.078	38.7 (26.0)	57.4 (34.7)	38.9 (27.3)	50.0 (29.5)	**30.8 (27.2)**	**48.4 (28.9)**	**49.3 (32.6)**	**30.8 (19.7)**	**49.1(29.0)**	**32.0(25.1)**	45.4 (30.0)	33.6 (26.2)	0.252
		*p*	0.176		0.341	0.557	0.064		0.197		**0.039**		**0.013**		**0.023**		0.177		0.061
	HI-S	M (SD) or *r*	30.7 (24.0)	39.6 (30.1)	0.106	0.070	33.0 (25.3)	54.6 (36.8)	33.0 (26.5)	47.0 (31.6)	**21.8 (20.8)**	**43.9 (31.1)**	**44.4 (30.5)**	**25.0 (23.4)**	**44.0(29.2)**	**26.0(24.8)**	39.0 (30.5)	31.3 (25.0)	0.066
		*p*	0.240		0.424	0.600	0.124		0.103		**0.004**		**0.010**		**0.017**		0.372		0.630
	HI-DL	M (SD) or *r*	34.8 (16.3)	44.7 (29.5)	0.083	-0.029	**37.1 (23.3)**	**62.8 (29.2)**	39.8 (24.7)	45.0 (29.2)	**25.6 (21.2)**	**50.2 (25.7)**	**48.0 (27.9)**	**31.4 (21.7)**	**47.5(25.4)**	**32.6(25.0)**	**46.6 (27.6)**	**26.6 (18.9)**	0.210
		*p*	0.101		0.534	0.827	**0.005**		0.512		**0.001**		**0.019**		**0.031**		**0.011**		0.120
	EC	M (SD) or *r*	29.0 (24.2)	37.5 (31.2)	-0.030	0.164	**31.0 (27.0)**	**52.8 (33.5)**	31.1 (27.5)	44.6 (31.7)	**20.8 (19.2)**	**37.5 (32.3)**	39.2 (30.6)	25.5 (25.4)	36.7(30.6)	32.0(27.7)	36.8 (32.4)	28.1 (23.0)	0.231
		*p*	0.276		0.819	0.215	**0.036**		0.127		**0.025**		0.081		0.550		0.334		0.087
	PD	M (SD) or *r*	40.3 (33.2)	43.9 (28.7)	0.065	-0.063	41.3 (29.9)	50.0 (32.5)	40.3 (29.8)	50.0 (31.4)	**25.0 (20.6)**	**51.1 (31.8)**	**50.0 (31.0)**	**31.5 (28.2)**	**50.4(29.9)**	**31.5(28.7)**	**48.7 (30.7)**	**21.9 (21.1)**	0.211
		*p*	0.664		0.625	0.637	0.428		0.297		**0.003**		**0.026**		**0.019**		**0.002**		0.118
	WP	M (SD) or *r*	50.0 (21.1)	55.6 (29.1)	0.032	0.117	**50.2 (25.7)**	**72.2 (22.4)**	51.9 (26.6)	58.9 (25.4)	46.8 (26.7)	57.3 (27.9)	58.3 (27.9)	47.0 (24.9)	59.6(23.8)	47.7(28.2)	58.3 (25.8)	47.9 (26.4)	0.358
		*p*	0.432		0.813	0.378	**0.019**		0.384		0.196		0.121		0.088		0.185		**0.007**
	PC	M (SD) or *r*	56.3 (22.1)	60.5 (27.2)	0.023	-0.198	58.0 (25.1)	63.9 (27.6)	59.4 (25.3)	57.1 (26.3)	53.5 (23.8)	64.8 (25.9)	**65.0 (25.1)**	**51.0 (25.5)**	62.1(24.9)	57.0(25.3)	**64.1 (24.0)**	**48.4 (24.5)**	0.296
		*p*	0.540		0.862	0.133	0.525		0.769		0.132		**0.046**		0.448		**0.034**		**0.027**
	FA	M (SD) or *r*	33.0 (34.8)	41.9 (40.0)	0.144	-0.034	34.5 (34.5)	61.1 (48.6)	35.6 (37.5)	48.2 (39.8)	**20.8 (31.2)**	**50.0 (40.5)**	**52.5 (40,.1)**	**20.0 (29.8)**	**49.2(39.9)**	**26.0(32.7)**	**48.0 (40.8)**	**20.3 (27.7)**	0.168
		*p*	0.388		0.277	0.798	0.149		0.281		**0.006**		**0.001**		**0.022**		**0.006**		0.215
	FDP	M (SD) or *r*	65.9 (41.9)	57.4 (38.6)	0.179	0.152	64.0 (37.9)	41.7 (46.8)	60.0 (41.4)	62.5 (35.0)	58.3 (40.2)	60.6 (41.0)	58.3 (41.7)	65.0 (38.9)	**71.9(37.4)**	**48.0(40.1)**	66.4 (40.4)	50.0 (37.6)	0.189
		*p*	0.433		0.174	0.249	0.121		0.839		0.850		0.545		**0.024**		0.169		0.162
	FIC	M (SD) or *r*	48.9 (34.0)	57.4 (38.1)	0.125	-0.010	51.0 (35.7)	72.2 (38.4)	51.7 (38.2)	62.5 (30.6)	**31.9 (30.7)**	**67.4 (34.5)**	**64.2 (33.3)**	**41.0 (39.4)**	**64.8(35.3)**	**41.0(36.0)**	**63.8 (34.7)**	**26.6 (30.9)**	0.145
		*p*	0.389		0.345	0.940	0.110		0.338		**0.001**		**0.022**		**0.015**		**0.001**		0.287
	ODC	M (SD) or *r*	39.8(36.7)	46.6(35.4)	0.078	0.067	42.0(35.2)	55.6(39.1)	**38.3(33.5)**	**62.5(37.7)**	**23.6(27.7)**	**54.5(37.7)**	**51.7(38.2)**	**32.0(31.9)**	51.6(35.9)	34.0(34.5)	50.0(36.8)	29.7(34.4)	0.262
		*p*	0.482		0.556	0.617	0.299		**0.026**		**0.002**		**0.046**		0.068		0.065		0.052
	SCQ total	M (SD) or *r*	37.9 (17.9)	45.8 (25.9)	0.081	-0.014	**39.9 (21.4)**	**59.3 (28.6)**	40.5 (22.8)	50.2 (24.9)	**30.0 (19.2)**	**49.8 (24.4)**	**49.7 (25.2)**	**32.9 (19.2)**	**49.5(7.9)**	**34.7(22.9)**	**47.8 (24.8)**	**31.4 (19.4)**	0.280
		*p*	0.172		0.541	0.918	**0.021**		0.181		**0.005**		**0.008**		**0.018**		**0.023**		**0.037**
SF12	PCS	M (SD) or *r*	44.77 (7.89)	40.51 (8.02)	-0.120	0.024	42.91 (8.02)	37.60 (8.01)	42.53 (8.42)	40.70 (7.43)	**45.65 (8.60)**	**39.73 (8.07)**	**40.01 (7.83)**	**44.59 (8.66)**	40.82(7.91)	43.68(8.71)	40.59 (8.04)	45.42 (8.98)	-0.293
		*p*	0.052		0.366	0.854	0.073		0.470		**0.018**		**0.044**		0.201		0.057		**0.028**
	MCS	M (SD) or *r*	**45.69 (7.37)**	**40.94 (8.71)**	-0.024	-0.299	43.29 (8.32)	39.50 (9.25)	**44.14 (8.01)**	**38.14 (8.69)**	**46.29 (6.21)**	**40.99 (9.76)**	42.20 (9.96)	43.32 (7.25)	41.06(8.81)	44.57(8.03)	42.23 (9.24)	44.77 (6.91)	-0.182
		*p*	**0.037**		0.860	**0.022**	0.220		**0.020**		**0.043**		0.641		0.126		0.328		0.179
CD-RISC-10		M (SD) or *r*	**27.9 (5.8)**	**22.4 (6.8)**	0.212	0.260	24.6 (7.0)	23.1 (7.0)	24.4 (7.3)	24.4 (6.0)	27.3 (7.2)	24.2 (5.3)	23.9 (7.0)	26.5 (5.6)	24.5(8.2)	24.5(5.5)	24.3 (7.5)	25.9 (5.2)	-0.077
		*p*	**0.003**		0.107	**0.046**	0.549		0.976		0.080		0.136		0.995		0.456		0.570

*Note*: M (SD) = mean (standard deviation); r = Pearson's correlation coefficients; In bold, 0.40 < correlations, *p*-value; In bold, p < 0.05; Abbreviations: see the note of Table 2.

We entered all variables that were significant or nearly-significant in the overall QoL scores (S-QoL 18 index, SCQ HI-TS, and total score) in the bivariate analysis into a multiple linear regression analysis, using a forced entry method for S-QoL and a stepwise method for SCQ. Significant regression equations were found (*F*(2,54) = 4.66, *p* = 0.014), with an *R*^*2*^ of 0.15) for S-QoL 18, (*F*(2, 46) = 7.46, *p* = 0.002, with an *R*^*2*^ of 0.25) for SCQ HI-TS, and (*F*(2,46) = 8.13, *p* = 0.001), with an *R*^*2*^ of 0.26) for the SCQ total score. The crude and standardized beta coefficients and the 95% confidence interval (CI) of the variables are presented in Table 6.

**Table 6:** Multiple regression analysis predicting patients' and family caregivers' QoL.

	S-QoL 18 index			
	Crude *B*	Standardized *B*	95% CI	*p*
(Constant)	51.96			
Occupation	11.37	0.31	1.73 to 20.52	0.021
Generation	10.48	0.29	1.21 to 19.8	0.028
	SCQ Humanistic impact			
	Crude *B*	Standardized *B*	95% CI	*p*
(Constant)	55.20			
Economic status	-18.43	-0.33	-33.4 to -3.50	0.017
Chronic disease	-16.83	-0.31	-31.2 to -2.4	0.023
	SCQ total			
	Crude *B*	Standardized *B*	95% CI	*p*
(Constant)	56.89			
Economic status	-17.33	-0.34	-30.7 to -4.0	0.012
Chronic disease	-15.59	-0.32	-28.5 to -2.7	0.019

*Note*: Dummy variables: Generation: pre = 0, post = 1; Occupation: paid = 1, unpaid = 0

Economic status: sufficient = 1, insufficient = 0; Chronic disease: yes = 0, no = 1

## Discussion

This study primarily aimed to create and validate a Khmer version of the QoL evaluation questionnaires: S-QoL 18 for patients with schizophrenia, and SCQ for family caregivers of patients with schizophrenia. Our results on internal consistency reliability and concurrent validity indicated that the S-QoL 18 Khmer version had good psychometric properties. The scores for resilience and autonomy, and the total score were negatively correlated with the severity of negative symptoms. These results were in accordance with previous studies [57–62]. Although it had a two-factor structure, unlike the original version [52], we suggest that the index (total score) is valid for use with Cambodian patients with schizophrenia to evaluate their overall degree of QoL. Regarding cultural considerations, we should mention that both the internal consistency reliability and concurrent validity were unsatisfactory for assessing the PsW domain, which consisted of three items to evaluate “how much the participant has difficulty concentrating, thinking straight”, “how much the participant feels cut-off from the outside world”, and “how much the participant has difficulty expressing his/her feelings”. These questions were originally generated from an analysis of interviews with patients with schizophrenia and validated in the Western population [35, 52]. In Cambodia, “thinking too much” is one of the common complaints relating, not only to past traumatic events, but also to current problems [1, 16]. With respect to emotional expressions, individuals who survived the KR regime tend to feel cowardly and behave submissively [63]; therefore, the first and third items above needed further cultural consideration to modify S-QoL 18 to a version more congruent with Cambodian culture.

Our results also indicated that the SCQ Khmer version had good psychometric properties. The original English version of the SCQ domain showed moderate correlations with SF-36 mental health score, but not physical health score, in the Western population [38]. This contrasted with our results, which aligned with the literature claiming that Cambodian individuals tend to develop physical symptoms in response to psychological distress [1, 16, 64]. Although the SCQ Khmer version had a single-factor structure, unlike the original English version, we suggest that the total score and SCQ HI-TS are valid for use with Cambodian family caregivers of patients with schizophrenia to evaluate their overall degree of QoL.

The second aim of this study was to elucidate the social determinants of QoL among patients with schizophrenia and their family caregivers. Among patients, the S-QoL 18 scores were gender-independent, in accordance with two other studies [59, 65]. Years of schooling did not correlate with QoL, and literacy significantly influenced only the SL dimension. Generally, higher education was related to better social functioning [66]; however, it did not seem to be a predominant factor for improving QoL in the Cambodian population. Regarding the generation gap, those who were born after the KR era showed better QoL and better family relationships, although their negative symptoms were more severe than those of people born during and before the KR era. The results implied that lower social functioning did not necessarily equate to higher subjective QoL. The patients with paid occupations showed significantly better QoL scores, which led us to believe that the critical factor for better QoL is paid work, which is congruent with previous studies [42, 61, 66–69]. As expected, patients with paid occupations showed lower PANSS-6 scores than those with unpaid occupations or who were unemployed. The economic status of households did not noticeably influence the patients’ QoL. These results suggested that family income was less important for patients than their financial productivity. The chlorpromazine-equivalent dose of antipsychotics used by patients was moderately negatively correlated with their physical well-being status. Since most of the patients used haloperidol and/or chlorpromazine in the present study, a lower dosage, rather than the use of atypical antipsychotics [68], seemed to be a key factor for better QoL. There were no correlations or significant differences between the socio-demographic factors and the CD-RISC-10 score; however, the patients with a paid occupation were more resilient according to the S-Qol 18 score, which was consistent with the results of previous studies [42, 61, 66–69]. DUP, which affects QoL at the first visit to a medical institution [70], did not influence any QoL domains among our sample patient population who had already received psychiatric treatment. In the multiple regression analysis, engaging in paid occupation predicted higher QoL in patients with schizophrenia. This factor was found to be related to better QoL in the bivariate analyses used by previous studies [42, 61, 66–69], and it was stated as a predictor of QoL by the multiple regression analysis used by one study in the Czech Republic [67]. Work plays a particularly important role in defining an individual’s identity [71], so the double discrimination due to unemployment and mental health problems is particularly harmful [72]. Our findings affirmed the need to provide interventions focusing on job skills training to generate positive effects on patients’ QoL. Being of the post-Khmer Rouge generation also predicted higher QoL for the patients. This result suggested that the KR regime had a long-term influence on how the patients felt about stressful events in daily life, which is compatible with several online media articles [19, 73]. However, this study did not ask about personal traumatic experiences; thus, further studies are needed to clarify the influence of the generation gap on QoL.

Among family caregivers, in particular, economic status, physical diseases, and kinship influenced QoL multidimensionally in the univariate analysis. Caregivers without paid occupations experienced greater difficulty in overall caregiving than those with paid occupations. It has been reported that caregivers’ employment status impacts their health status [74], but the economic status of households, rather than employment status, seemed to be an important factor for our caregiver sample. This may reflect the family members’ possible commitment to agricultural labor or family-operated independent businesses (the two leading occupations). Kinship, or a caregiver’s role in the family, was reported to affect the caregiver’s perceptions of the burden of care, but the results of previous studies were inconsistent [75]. Our results showed that the QoL of the parents of patients was lower than that of the caregivers with other family relationships, which was congruent with several studies [76, 77]. Patients’ dependence on parents inevitably makes the parents feel worried about who will care for the patients in the future. Old age often causes physical problems [33] and half of the caregivers in our participants had chronic disease. Gender was not correlated with any SCQ dimensions, and this result corresponded to the results of several other studies [78–80]. In the multiple regression analysis, two aspects, including adequate household economic status and the absence of chronic disease, predicted higher QoL for family caregivers. These results were in accordance with those of a previous study reporting that the household income and physical health of caregivers were predictors of their QoL [81]. In countries where support for continuous treatment and long-term medication fees, community and health resources, and the availability of medication were scarce, previous studies showed that household income was of the utmost concern to caregivers [82]. Living with chronic disease makes caregiving responsibilities more tiring and necessitates the caregivers allocating more money to treatment and medication fees from the family budget. These results suggested that financial support for the households, including help with hospital fees for patients and caregivers with chronic illnesses, and home care for patients to reduce the caregiver burden, are needed to improve the caregiver’s QoL.

The main limitations of the present study were its cross-sectional design, relatively small sample size, and the inability to analyze the relationships between patients and their own caregivers due to insufficient patient–caregiver dyads. We were also unable to perform the step-by-step linguistic validation process recommended by the Mapi Research Institute for S-QoL 18 and SCQ [83], due to resource limitations. The structures of S-QoL 18 and SCQ differed from the original versions in our participants. In addition, the coefficient of determination for the S-QoL index was relatively low at 0.15. These results may indicate that QoL is multifactorial and individual-specific, which supports the evidence claiming that financial interventions in low- and middle-income countries are not always successful [84]. In order to overcome these limitations, it would be advisable to conduct research using these newly created Khmer versions of S-QoL 18 and SCQ total or SCQ HI-TS with larger sample groups in various clinical settings in Cambodia.

## Conclusions

This first investigation of the QoL of patients with schizophrenia and their family caregivers in Cambodia indicated the following:

1. The newly translated Khmer version of S-QoL 18, and SCQ total and SCQ HI-TS, are relatively good psychometric tools for evaluating the QoL of patients with schizophrenia and their family caregivers in Cambodian clinical settings. They can be used for further research into patients’ and caregivers’ needs to improve their QoL in Cambodia.
2. Engaging in paid work, or being of the post-KR generation, results in higher QoL for patients, while having low household economic status, or being affected by chronic disease, leads to lower QoL for family caregivers.

These findings are useful for helping community mental health professionals and aid organizations to create better programs to lessen the patient and caregiver burden in Cambodia. Further investigation is necessary to develop practical projects that will improve patients’ and caregivers’ QoL in various clinical settings in Cambodia.

## Supporting information

S1 Table (PDF)

S2 Table (PDF)
