# Correction: Quality of life and its social determinants for patients with schizophrenia and family caregivers in Cambodia

**DOI:** 10.1371/journal.pone.0232434

**Published:** 2020-04-23

**Authors:** The PLOS ONE Staff

There are errors in the RFa column of Table 3. The publisher apologizes for these errors. Please see the correct Table 3 here.

**Table 3:** Correlations between S-QoL 18 or SCQ scores and SF-12, CD-RISC 10, and between S-QoL 18 and PANSS-6 scores.

		S-QoL18 domains													
		PsW	SE	RFa	RFr	RE	PhW	AU	SL	Index					
SF-12	PCS	0.125	0.301*	0.062	0.269*	0.174	0.513**	0.213	0.208	0.350**					
	MCS	0.081	0.610**	0.375**	0.430**	0.493**	0.547**	0.431**	0.348**	0.609**					
CD-RISC-10		0.274*	0.637**	-0.019	0.492**	0.545**	0.421**	0.417**	0.354**	0.571**					
PANSS-6	Positive	0.017	-0.346**	-0.182	-0.372**	-0.327*	-0.278*	-0.505**	-0.377**	-0.439**					
	Negative	0.127	-0.409**	-0.130	-0.359**	-0.544**	-0.241	-0.486**	-0.343**	-0.438**					
	Total	0.089	-0.440**	-0.178	-0.422**	-0.513**	-0.298*	-0.572**	-0.415**	-0.507**					
		SCQ domains													
		HI-TS	HI-P	HI-E	HI-S	HI-DL	EC	PD	WP	PC	FA	FDP	FIC	ODC	SCQ total
SF-12	PCS	-0.497**	-0.543**	-0.416**	-0.300*	-0.560**	-0.252	-0.443**	-0.412**	-0.205	-0.294*	-0.284*	-0.442**	-0.470**	-0.501**
	MCS	-0.394**	-0.339**	-0.328*	-0.426**	-0.391**	-0.321*	-0.284*	-0.285*	-0.106	-0.295*	-0.158	-0.377**	-0.352**	-0.398**
CD-RISC-10		-0.318*	-0.333*	-0.282*	-0.267*	-0.301*	-0.245	-0.231	-0.134	-0.121	-0.213	0.174	-0.141	-0.195	-0.297*

*Note*: Pearson’s correlation coefficients; in bold, 0.40 < absolute value of correlations

* p < 0.05

** p < 0.01

Abbreviations: see the note of Table 2.
