# Supplementary material for: Quality of life and its social determinants for patients with schizophrenia and family caregivers in Cambodia
Source: PLoS One. 2020 Mar 4;15(3):e0229643. doi: 10.1371/journal.pone.0229643 (PMC7055908; doi:10.1371/journal.pone.0229643)
Supplement: S1 Table — (PDF) [file pone.0229643.s001.pdf]

S1 Table. Supporting information of socio-demographic factors of patients associated with S-QoL 18, SF-12, CD-RISC 10, and PANSS-6

			Marital status		Residence		No of person per household	Duration of patient's illness	DUP
			Married	Other	Urban	Rural			
S-QoL 18	PsW	M (SD) or *r*	54.7 (24.1)	64.4 (25.9)	64.2 (23.7)	53.1 (27.8)	-0.149	-0.147	-0.160
		p		0.157		0.749	0.281	0.276	0.238
	SE	M (SD) or *r*	64.7(23.4)	66.7(27.1)	65.3(28.5)	67.1(18.3)	-0.094	0.038	-0.049
		p		0.773		0.681	0.498	0.780	0.719
	RFa	M (SD) or *r*	72.9 (31.0)	79.2 (21.5)	75.9 (26.6)	78.3 (23.9)	-0.041	-0.090	-0.123
		p		0.397		0.783	0.769	0.506	0.365
	RFr	M (SD) or *r*	57.1 (27.4)	55.2 (24.2)	53.1 (25.9)	61.8 (23.4)	-0.031	0.040	0.003
		p		0.786		0.424	0.826	0.766	0.983
	RE	M (SD) or *r*	63.0 (28.5)	70.8 (22.7)	67.3 (24.4)	68.9 (27.3)	-0.006	0.066	-0.143
		p		0.276		0.234	0.965	0.628	0.294
	PhW	M (SD) or *r*	51.1 (40.0)	58.7 (30.6)	52.8 (32.5)	61.8 (27.8)	0.171	-0.143	-0.089
		p		0.365		0.347	0.217	0.288	0.513
	AU	M (SD) or *r*	63.6(32.8)	70.5 (24.5)	64.1 (29.3)	75.7 (23.7)	-0.021	0.026	-0.092
		p		0.392		0.682	0.880	0.847	0.499
	SL	M (SD) or *r*	63.0 (29.6)	46.2 (30.0)	47.2 (30.0)	64.5 (28.6)	-0.160	0.020	-0.122
		p		0.037		0.438	0.249	0.884	0.371
	Index (total)	M (SD) or *r*	61.3 (21.4)	63.9 (16.6)	61.2 (18.6)	66.4 (18.2)	-0.057	-0.037	-0.141
		p		0.590		0.950	0.683	0.782	0.300
SF12	PCS	M (SD) or *r*	45.3 (10.9)	44.9 (10.3)	44.4 (11.2)	46.4 (8.5)	0.008	0.158	0.241
		p		0.900		0.557	0.956	0.242	0.073
	MCS	M (SD) or *r*	40.6 (8.4)	43.6 (10.0)	43.4 (9.6)	40.6 (9.1)	0.035	-0.016	-0.063
		p		0.239		0.174	0.799	0.906	0.646
CD-RISC-10		M (SD) or *r*	22.1 (7.0)	22.0(7.0)	21.7 (7.1)	22.7 (6.7)	-0.103	0.200	0.102
		p		0.933		0.870	0.457	0.136	0.455
PANSS-6	Positive	M (SD) or *r*	5.9(3.3)	6.8 (3.7)	6.9 (4.0)	5.5 (2.2)	0.126	-0.026	-0.027
		p		0.365		0.657	0.366	0.846	0.845
	Negative	M (SD) or *r*	5.7 (3.3)	7.2 (4.6)	7.3 (4.6)	5.2 (2.7)	0.001	-0.196	-0.048
		p		0.171		0.489	0.996	0.143	0.723
	Total	M (SD) or *r*	11.6 (6.4)	14.0 (6.8)	14.1 (7.4)	10.7 (4.1)	0.067	-0.138	-0.044
		p		0.180		0.497	0.631	0.307	0.746

Note: M (SD) = mean (standard deviation); r = Pearson's correlation coefficients;

PsW = Psychological well-being; SE = Self-esteem; RFa = Family relationships; RFr = Relationships with friends; RE = Resilience;

PhW = Physical well-being; AU = Autonomy; SL = Sentimental life;

PCS = Physical component summary; MCS = Mental component summary
