# Supplementary material for: Quality of life and its social determinants for patients with schizophrenia and family caregivers in Cambodia
Source: PLoS One. 2020 Mar 4;15(3):e0229643. doi: 10.1371/journal.pone.0229643 (PMC7055908; doi:10.1371/journal.pone.0229643)
Supplement: S2 Table — (PDF) [file pone.0229643.s002.pdf]

S2 Table. Supporting information of socio-demographic factors of family caregivers associated with SCQ, SF-12, and CD-RISC 10

			Generation		Marital status		Residence		Duration of caregiving	No of person per household	Duration of patient's illness	DUP
			Pre-KR	Post-KR	Married	Other	Urban	Rural				
SCQ	HI-TS	M (SD) or *r*	41.5 (26.2)	36.3 (25.4)	37.8 (27.2)	45.0 (22.0)	40.5 (28.3)	39.3 (23.7)	0.055	0.032	-0.021	0.061
		p		0.482		0.339		0.853	0.703	0.828	0.893	0.703
	HI-P	M (SD) or *r*	39.2 (32.1)	36.1 (30.1)	36.5 (33.3)	42.6 (26.0)	39.3 (31.8)	37.4 (31.3)	0.039	0.115	-0.008	0.074
		p		0.728		0.500		0.816	0.785	0.426	0.959	0.646
	HI-E	M (SD) or *r*	42.9 (29.1)	38.4 (25.7)	38.8 (28.6)	48.3 (26.0)	43.0 (29.6)	40.2 (26.9)	0.031	0.018	0.009	0.023
		p		0.577		0.241		0.703	0.828	0.904	0.954	0.888
	HI-S	M (SD) or *r*	39.0 (29.1)	30.1 (25.6)	35.1 (29.5)	39.2 (25.1)	37.5 (31.8)	35.2 (24.9)	0.084	-0.062	0.029	0.186
		p		0.265		0.617		0.758	0.558	0.671	0.856	0.243
	HI-DL	M (SD) or *r*	42.6 (24.7)	37.5 (28.3)	39.0 (26.4)	45.9 (24.0)	40.2 (29.6)	41.8 (22.1)	0.064	0.042	-0.100	0.003
		p		0.491		0.360		0.814	0.657	0.770	0.528	0.985
	EC	M (SD) or *r*	33.2 (30.1)	36.8 (26.6)	32.1 (29.0)	39.7 (28.7)	29.5 (31.6)	38.7 (25.9)	-0.155	-0.073	0.018	-0.155
		p		0.665		0.367		0.222	0.277	0.616	0.912	0.334
	PD	M (SD) or *r*	43.6 (28.8)	40.3 (33.9)	39.6 (32.1)	50.0 (24.2)	42.9 (31.4)	42.3 (29.5)	0.231	-0.038	-0.101	-0.117
		p		0.701		0.233		0.948	0.103	0.791	0.523	0.466
	WP	M (SD) or *r*	52.6 (25.4)	55.6 (29.0)	50.2 (26.3)	61.8 (25.2)	55.1 (29.1)	52.2 (24.0)	-0.027	-0.015	-0.189	-0.066
		p		0.699		0.127		0.675	0.852	0.916	0.231	0.683
	PC	M (SD) or *r*	57.9 (27.2)	61.1 (21.0)	60.4 (24.4)	55.1 (28.0)	58.0 (26.0)	59.7 (25.1)	0.060	-0.175	0.026	-0.198
		p		0.660		0.474		0.806	0.677	0.225	0.872	0.214
	FA	M (SD) or *r*	39.6 (38.7)	36.1 (37.6)	36.9 (38.7)	42.6 (37.3)	42.9 (40.2)	34.7 (36.3)	0.034	-0.003	0.016	0.044
		p		0.747		0.604		0.415	0.811	0.986	0.919	0.784
	FDP	M (SD) or *r*	65.2 (39.1)	50.0 (40.2)	63.1 (42.1)	54.4 (33.3)	67.0 (37.9)	54.8 (41.0)	0.319	-0.080	-0.302	-0.257
		p		0.177		0.408		0.245	0.022	0.582	0.052	0.104
	FIC	M (SD) or *r*	55.5 (37.3)	51.4 (35.8)	50.0 (38.7)	64.7 (29.4)	55.4 (38.7)	53.2 (35.2)	0.089	-0.023	-0.162	-0.070
		p		0.696		0.164		0.825	0.535	0.873	0.305	0.662
	ODC	M (SD) or *r*	43.9(35.3)	44.4(37.9)	41.7 (35.6)	50.0 (36.4)	49.1 (38.8)	39.5 (32.8)	-0.059	-0.090	0.097	0.024
		p		0.958		0.422		0.308	0.678	0.535	0.539	0.882
	SCQ total	M (SD) or *r*	43.5 (23.2)	41.4 (24.6)	41.0 (24.4)	47.3 (20.9)	43.2 (26.4)	42.5 (20.9)	0.049	-0.016	-0.049	-0.019
		p		0.754		0.356		0.916	0.732	0.913	0.756	0.905
SF12	PCS	M (SD) or *r*	40.91 (8.16)	44.80 (7.76)	42.31 (7.89)	41.56 (9.08)	43.10 (8.74)	41.19 (7.65)	-0.084	-0.029	0.190	0.048
		p		0.092		0.751		0.374	0.560	0.842	0.227	0.768
	MCS	M (SD) or *r*	42.03 (8.66)	44.27 (8.13)	43.29 (8.17)	41.28 (9.34)	41.94 (9.08)	43.41 (8.01)	0.135	0.027	0.178	-0.195
		p		0.356		0.414		0.511	0.346	0.852	0.259	0.221
CD-RISC-10		M (SD) or *r*	24.7 (6.0)	23.8 (8.9)	25.2 (6.5)	22.5 (7.8)	24.6 (6.3)	22.4 (7.0)	0.062	-0.284	-0.275	-0.319
		p		0.722		0.190		0.018	0.665	0.045	0.078	0.042

M (SD) = mean (standard deviation); r = Pearson's correlation coefficients;
KR = Khmer Rouge; HI-TS = Humanistic impact - Total Scores; HI-P = Humanistic impact - Physical; HI-E = Huministic impact - Emotional; HI-S = Humanistic impact - Social;
HI-DL = Humanistic impact - Daily life; EC = Exhaustion with caregiving; PD = Patient dependence; WP = Worries for the patient; PC = Perception of caregiving; FA = Feeling alone;
FDP = Financial dependence of the patient; FIC = Financial impact of caregiving; ODC = Overall difficulty of caregiving;
PCS = Physical component summary; MCS = Mental component summary
